# Supplementary material for: Causal interplay between lactose intolerance and gut microbiota: a combined bidirectional Mendelian randomization and in vivo validation study
Source: Front Nutr. 2026 Jun 1;13:1803337. doi: 10.3389/fnut.2026.1803337 (PMC13265576; doi:10.3389/fnut.2026.1803337)
Supplement: Supplementary file 1 [file Data_Sheet_1.zip › supplementary materials/Forward/leave one out cross validation/ebi-a-GCST90027507.finngen_R12_E4_LACTONAS.pdf]

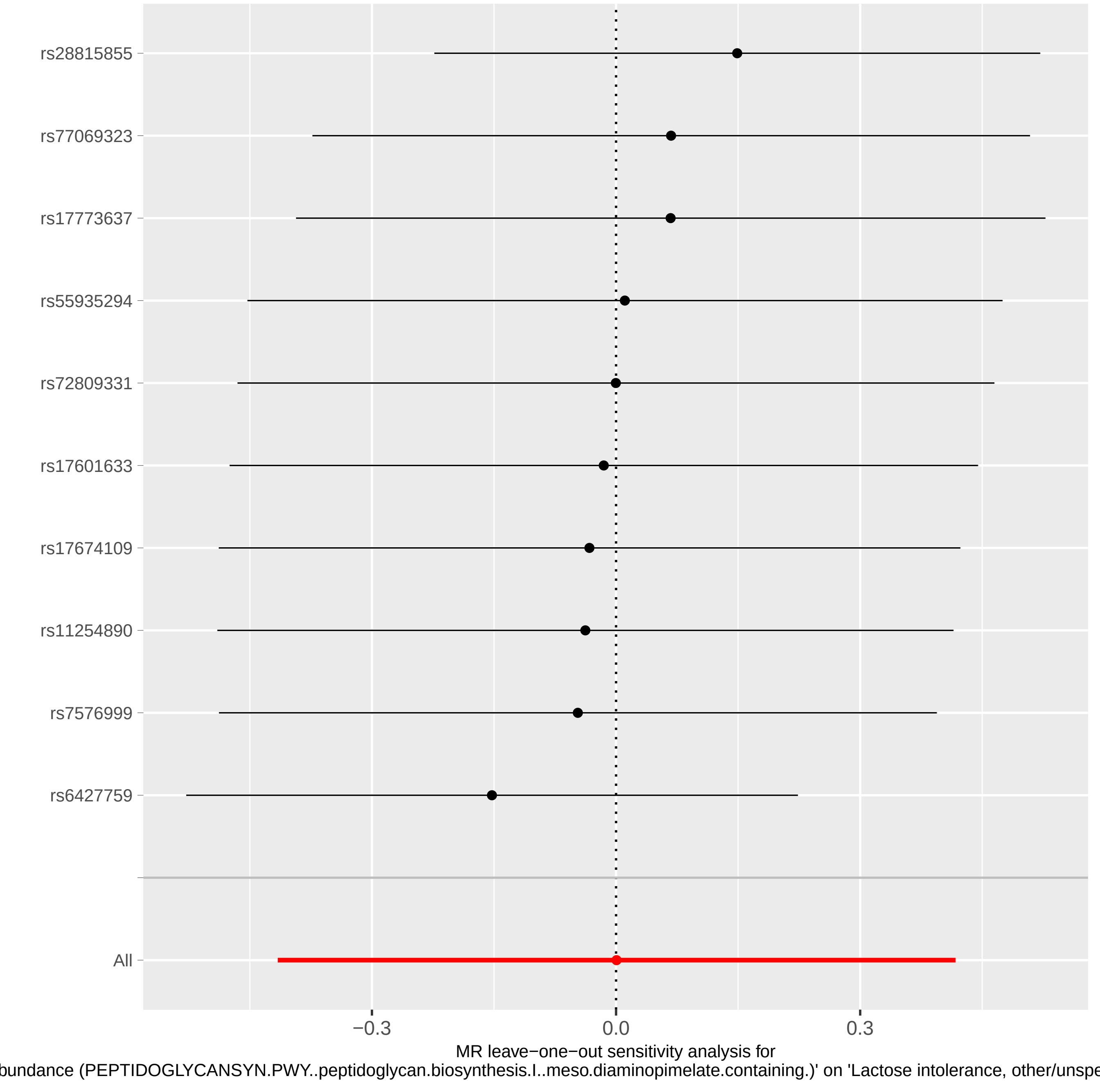
rs28815855
rs77069323
rs17773637
rs55935294
rs72809331
rs17601633
rs17674109
rs11254890
rs7576999
rs6427759
All
−0.3
0.0
0.3
MR leave−one−out sensitivity analysis for
bundance (PEPTIDOGLYCANSYN.PWY..peptidoglycan.biosynthesis.I..meso.diaminopimelate.containing.)' on 'Lactose intolerance, other/unspe
